# Controllable Chemical Vapor Deposition Synthesis and Second-Harmonic Generation of Rhombohedral Cr_2_S_3_

**DOI:** 10.3390/nano16171088

**Published:** 2026-08-31

**Authors:** Danliang Zhang, Peiran Li, Sihan Liu, Qing Ye, Ying Chen

**Affiliations:** 1 Key Laboratory of Low-Dimensional Quantum Structures and Quantum Control of Ministry of Education, Hunan Research Center of the Basic Discipline for Quantum Effects and Quantum Technologies, School of Physics and Electronics, Hunan Normal University, Changsha 410081, China; 2 State Key Laboratory of Pulsed Power Laser Technology, Key Laboratory of Electronic Restriction of Anhui Province, National University of Defense Technology, Advanced Laser Technology Laboratory of Anhui Province, Hefei 230037, China

**Keywords:** chemical vapor deposition, rhombohedral Cr_2_S_3_, non-van der Waals, second-harmonic generation, polarization

## Abstract

Non-layered two-dimensional (2D) chromium-based chalcogenides have garnered significant attention due to their distinctive magnetic, electronic, and optical properties. In this work, we report the controllable synthesis of high-quality rhombohedral Cr_2_S_3_ nanosheets on mica substrates via an atmospheric pressure chemical vapor deposition (CVD) strategy. The as-grown rhombohedral Cr_2_S_3_ nanosheets exhibit pronounced optical second-harmonic generation (SHG) signals, which show thickness-dependent enhancement and a strong dependence on both the linear excitation and detection polarization configurations. Most notably, temperature-dependent SHG measurements reveal a distinct inflection point near the Néel temperature (T_N_ ≈ 120 K), where the SHG intensity exhibits a sharp enhancement upon cooling below T_N_. This behavior arises from the additional magnetic dipole contributions activated by magnetic ordering, establishing SHG as a sensitive probe of magnetic phase transitions in non-layered 2D magnetic materials. Furthermore, circularly polarized SHG signals exhibit nearly 100% circular polarization at low temperatures. This work systematically elucidates the nonlinear optical response characteristics of rhombohedral Cr_2_S_3_ and their correlation with magnetic ordering transitions, laying an experimental foundation for the application of 2D non-van der Waals magnetic materials in nonlinear optoelectronics and spin-optoelectronic devices.

## 1. Introduction

Two-dimensional (2D) magnetic materials have attracted extensive interest owing to their unique magnetic [1,2,3], electronic [3,4,5,6], and optical properties [1,7] at the 2D limit, which hold great promise for device applications in spintronics, valleytronics, and nonlinear optics. Among these, chromium-based chalcogenides (e.g., Cr_2_S_3_, Cr_2_Se_3_, and Cr_2_Te_3_) are of particular interest due to their rich physical phenomena, including temperature-dependent magnetic states [8,9,10,11], crystalline spin glass states [12], air-stable ferromagnetism [13,14], and other interesting physical properties [8,15,16,17,18,19]. Cr_2_S_3_, as a typical non-layered chromium-based sulfide [8,9,10,15,17,20], exists in two primary polymorphs: the thermodynamically stable rhombohedral phase (space group $R\bar{3}$) and the metastable trigonal phase (space group $p\bar{3}1c$). The rhombohedral phase of Cr_2_S_3_ features a layered structure composed of CrS_2_ layers intercalated with partially occupied Cr layers, forming a non-van der Waals material with strong in-plane and out-of-plane covalent bonding. Rhombohedral Cr_2_S_3_ exhibits large magnetoresistance and ferrimagnetic properties, with a magnetic transition temperature (T_N_) of 120 K [8,9,10]. Moreover, rhombohedral Cr_2_S_3_ has been confirmed to exhibit out-of-plane centrosymmetry breaking via vertical piezoresponse force microscopy (PFM) characterization [21], providing a structural basis for investigating its nonlinear optical responses.

Second-harmonic generation (SHG) is a second-order nonlinear optical process that is strictly forbidden in centrosymmetric media under the electric dipole approximation [22,23,24]. The SHG response is described by the second-order polarization: $P_{i}^{\left(2\right)}(2\omega )=\varepsilon_{0}\sum_{ji}\chi_{ijk}^{\left(2\right)}(2\omega )E_{j}(\omega )E_{k}(\omega )$, where *χ_ijk_*^(2)^ is the second-order nonlinear susceptibility tensor, which vanishes identically for materials with inversion symmetry. As a symmetry-sensitive probe, SHG has been widely employed to investigate crystal structures [25,26], ferroelectric domains [27,28], magnetic ordering [29,30], and interfacial properties in various materials [25,31]. For 2D materials [32,33,34,35,36], SHG is highly sensitive to structural symmetry, and non-centrosymmetric structures can generate strong SHG signals, whereas centrosymmetric structures exhibit zero SHG signal under the electric dipole approximation. Recent studies have revealed that even in centrosymmetric bulk materials, surface symmetry breaking can induce appreciable SHG responses [21]. Notably, SHG signals have been observed in trigonal phase Cr_2_S_3_ nanosheets [21], originating from surface sulfur vacancies and dangling bonds that break inversion symmetry. Furthermore, magnetic order in two-dimensional magnetic materials that simultaneously breaks both spatial and time-reversal symmetries can induce a strong second-harmonic generation response [29,30]. Although SHG studies on 2D magnetic materials have been reported, systematic investigations of SHG properties in rhombohedral Cr_2_S_3_, particularly the relationship between these phenomena and magnetic order, remain unclear.

In this work, we synthesize high-quality rhombohedral Cr_2_S_3_ nanosheets on freshly cleaved mica substrates via the CVD method. The crystal structure is systematically confirmed by Raman spectroscopy and scanning electron microscopy (SEM). Power-dependent SHG measurements verify the second-order nonlinear optical nature of the Cr_2_S_3_ nanosheets, with the SHG intensity showing a monotonically increasing trend as the thickness increases. We measured the linearly polarized SHG signals of Cr_2_S_3_ as a function of excitation light and detection conditions. Furthermore, temperature-dependent SHG spectroscopy and circularly polarized SHG measurements establish the correlation between nonlinear optical responses and magnetic ordering transitions. This work systematically elucidates the nonlinear optical response characteristics of rhombohedral Cr_2_S_3_ and their coupling to magnetic ordering transitions, laying a foundation for the application of 2D non-van der Waals magnetic materials in nonlinear optoelectronics and spin-optoelectronic devices.

## 2. Experimental Methods

### 2.1. CVD Synthesis of Rhombohedral Cr_2_S_3_ Nanosheets

**Figure 1:**
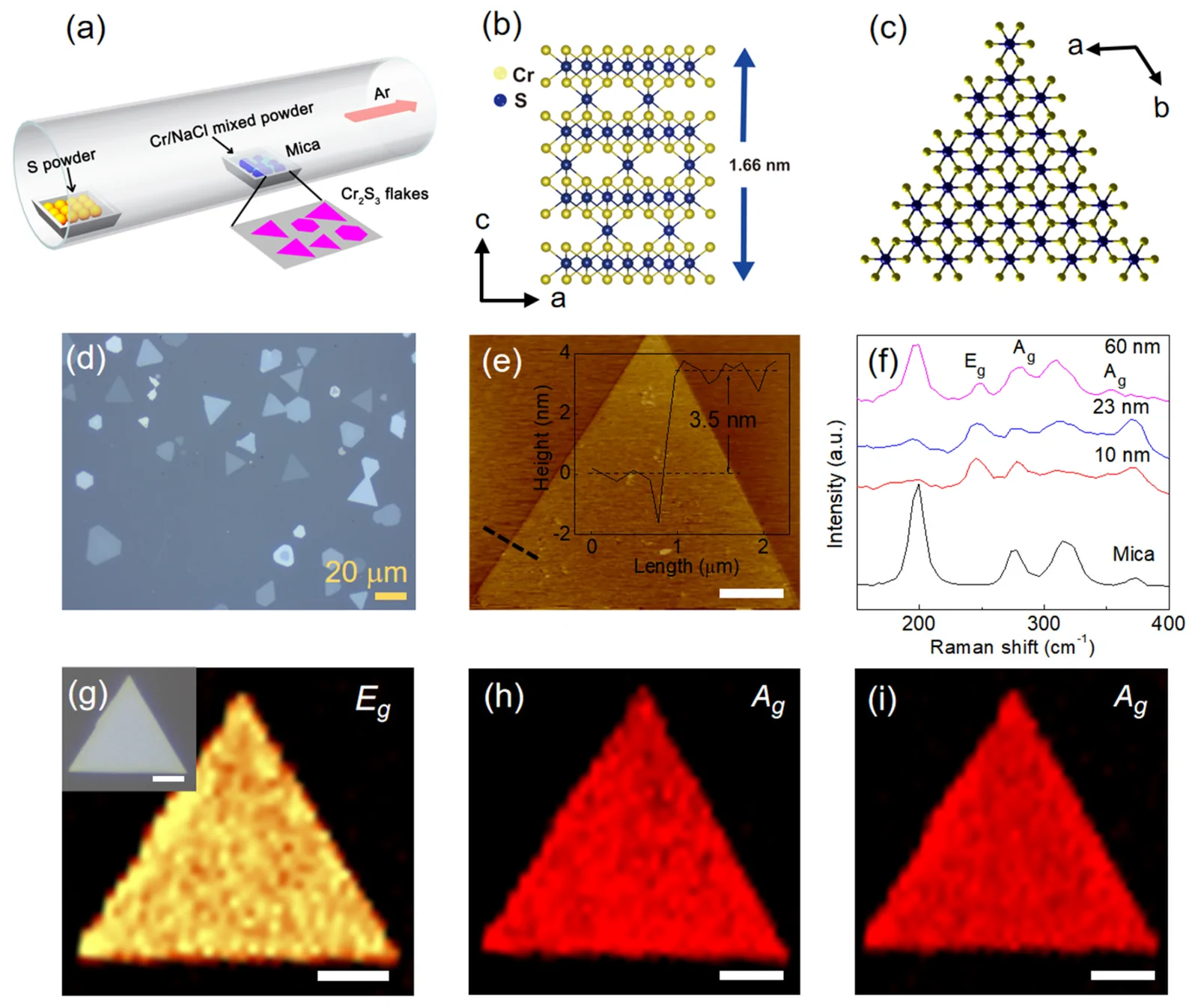
CVD growth and structural characterization of Cr_2_S_3_ nanosheets. (**a**) Schematic view of the Cr_2_S_3_ CVD growth process, yellow: S powder, blue: Cr, gray: NaCl powder. (**b**,**c**) Side-view and top-view crystal structures of rhombohedral Cr_2_S_3_. (**d**) Optical microscopy image of large-area Cr_2_S_3_ nanosheets on mica. Scale bar: 20 μm. (**e**) AFM image of a Cr_2_S_3_ nanosheet (scale bar: 10 μm). Inset: height profile along the black dashed line, showing a thickness of approximately 3.5 nm. (**f**) Raman spectra of Cr_2_S_3_ nanosheets with different thicknesses and the mica substrate. (**g**–**i**) Raman mapping of the out-of-plane *E_g_* mode (at 247 cm^−1^) and in-plane *A_g_* modes (at 280 and 364 cm^−1^). The inset in (**g**) shows the corresponding bright-field optical image. Scale bar: 10 μm.

Rhombohedral Cr_2_S_3_ nanosheets were synthesized via CVD in a single-zone tube furnace equipped with a quartz tube of 25 mm inner diameter (Figure 1a). A powdered mixture of NaCl (99.99%, Alfa, Thermo Fisher Scientific Inc., Heysham, Lancashire, UK) and chromium metal powder (Cr, 99%, Alfa, Thermo Fisher Scientific Inc., Heysham, Lancashire, UK) in a weight ratio of 10:1 was loaded into a ceramic boat as the reaction precursor. NaCl was employed to lower the melting point of Cr powder (melting point is 1857 °C), reduce the energy barrier, and enhance the reaction rate [37]. Freshly cleaved mica sheets (KMg_3_AlSi_3_O_10_F_2_, 15 × 15 mm) were placed face-down directly above the Cr source as the growth substrate. A ceramic boat containing sulfur powder (S, 99.9%) was positioned upstream in the tube furnace. Before starting the reaction, the tube was purged with 500 sccm Ar for 10 min to remove residual oxygen. Subsequently, the Ar flow rate was adjusted to 60 sccm, and the furnace temperature was ramped from room temperature to 850–900 °C over approximately 30 min. The reaction was maintained for 3–5 min, after which the system was rapidly cooled to room temperature.

### 2.2. Structural and Optical Characterization

Optical microscopy (Axio Scope A1, Zeiss, Oberkochen, Germany) was employed to observe and record the surface morphology of the as-grown Cr_2_S_3_ nanosheets. Atomic force microscopy (AFM, Bruker Multimode 8, Bruker Corporation, Billerica, MA, USA) was used for morphology and thickness measurements. Scanning electron microscopy (SEM) imaging and energy-dispersive X-ray spectroscopy (EDS) (SIGMA HD, Zeiss, Oberkochen, Germany) were performed to determine the chemical composition and elemental ratios. Raman spectroscopy (WITec alpha 300, WITec GmbH, Ulm, Germany, 532 nm excitation) was utilized for phase and compositional analysis.

### 2.3. SHG Measurements

SHG measurements were performed using a confocal microscope system (WITec alpha-300, WITec GmbH, Ulm, Germany). A femtosecond laser (Spectra-Physics Mai Tai, Santa Clara, CA, USA, pulse width 100 fs, repetition rate 80 MHz) served as the excitation source. The fundamental beam was focused onto the sample through a 50× objective (0.75 NA, Zeiss, Jena, Germany). The SHG signal was collected in reflection geometry by the same objective, filtered by a 400 nm bandpass filter, and detected by a spectrometer (UHTS300, WITec GmbH, Ulm, Germany) equipped with a CCD. For linear polarization-resolved measurements, a half-wave plate (λ/2) was placed in the excitation path to rotate the polarization state of the incident beam, and a polarizer was placed in the detection path to select the polarization state of the SHG signal.

For circular polarization SHG measurements, the femtosecond laser first passed through a linear polarizer (LP1, GTH10M-AM-A, Thorlabs, Newton, NJ, USA) to obtain high-quality linearly polarized light, which was then directed through a rotatable broadband quarter-wave plate (λ/4, AQWP05M-600, Thorlabs, Newton, NJ, USA). By precisely adjusting the optical axis of the λ/4 plate, the linearly polarized light could be converted to left-handed (*σ*^−^) or right-handed (*σ*^+^) circularly polarized light. In the detection path, the collected SHG signal passed through a λ/4 plate and a linear polarizer (LP2, WP25M-UB, Thorlabs, Newton, NJ, USA), followed by a filter to remove the fundamental light, and was then recorded by the spectrometer and CCD. By rotating LP2, either *σ*^−^ or *σ*^+^ SHG signals could be selectively detected. The specific optical layout is presented in Supplementary Note S1. For temperature-dependent measurements, the sample was located in the cryostat (Cryo Industries of America, Manchester, NH, USA) with temperature control ranging from 10 K to 300 K.

## 3. Results and Discussion

Figure 1a presents a schematic of the CVD growth process for high-quality rhombohedral Cr_2_S_3_ nanosheets. The crystal structure of Cr_2_S_3_ single crystals can be viewed as alternating stacked edge-sharing and independent CrS_6_ octahedral layers, with a fraction of chromium atoms intercalated within the gaps between CrS_2_ layers. This a-b-c stacking sequence along the c-direction gives rise to a crystal structure with $R\bar{3}$ space group, with a single-unit-cell thickness of 16.6 Å (Figure 1b) and lattice constants of a = b = 5.94 Å and c = 16.67 Å (Figure 1c). Figure 1d displays optical microscopy images of as-grown Cr_2_S_3_ nanosheets on mica, where different thicknesses exhibit distinct color contrasts. AFM image and corresponding height profile confirm the flat surface of the CVD-grown Cr_2_S_3_ nanosheets (Figure 1e), with the thinnest flakes measuring approximately 3.5 nm, corresponding to about two unit-cell thicknesses at the 2D limit. Raman spectra exhibit characteristic peaks at 247, 280, and 364 cm^−1^ (Figure 1f), corresponding to the out-of-plane *E_g_* and in-plane *A_g_* vibrational modes of rhombohedral Cr_2_S_3_. As the nanosheet thickness increases from 10 nm to 60 nm, the intensity of these Cr_2_S_3_ vibrational peaks gradually increases [8,20]. Combined SEM and EDS analysis confirms the phase purity of the synthesized rhombohedral Cr_2_S_3_ (Supplementary Note S2). Raman mapping reveals uniform signal distribution across the nanosheet (Figure 1g–i), confirming the phase purity and thickness uniformity of the samples. The controllable synthesis of high-quality non-layered 2D Cr_2_S_3_ provides a solid material foundation for subsequent SHG investigations.

**Figure 2:**
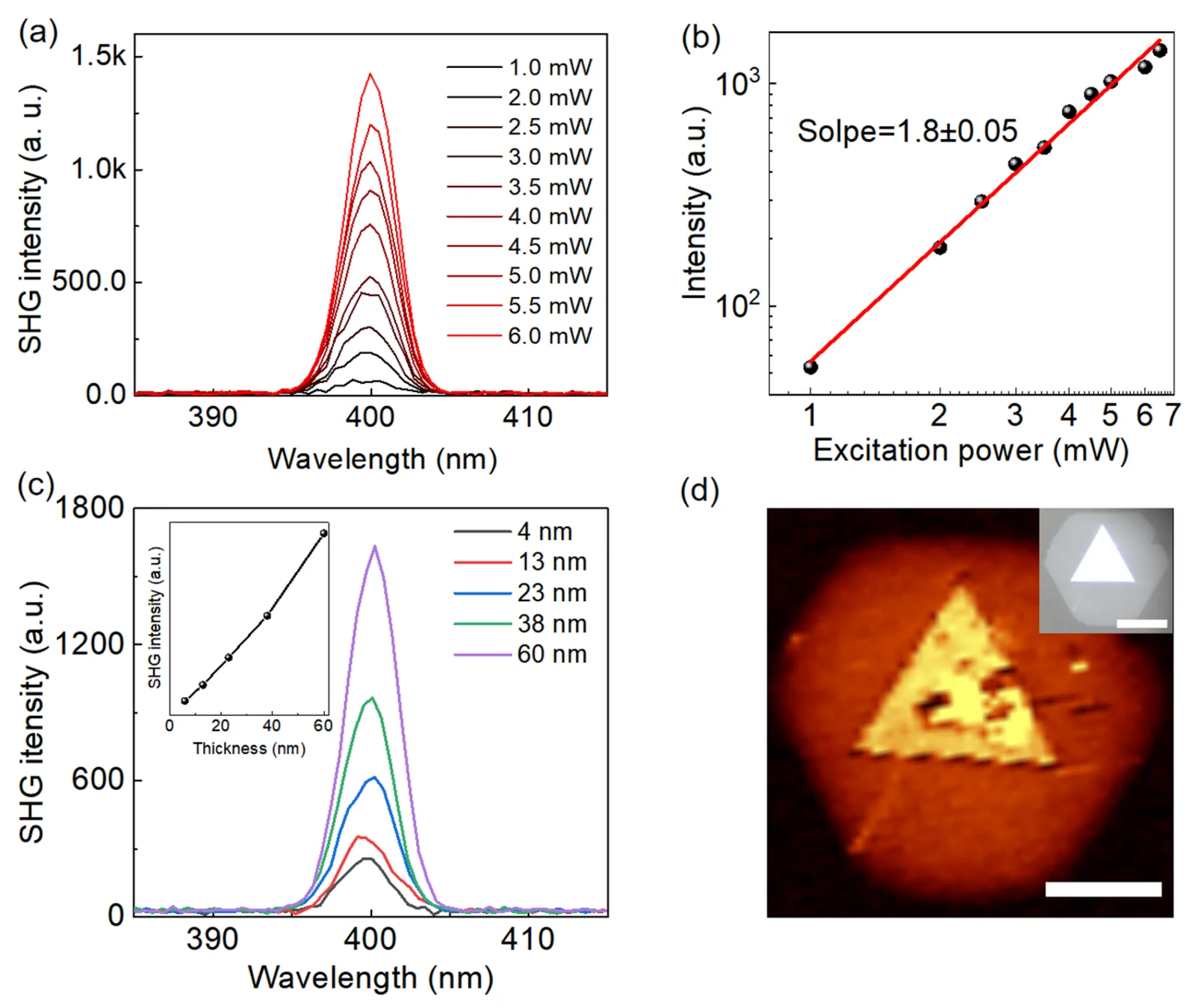
Power- and thickness-dependent SHG characterization of Cr_2_S_3_ nanosheets. (**a**) SHG spectra of Cr_2_S_3_ nanosheets under different excitation powers. (**b**) Double-logarithmic plot of SHG peak intensity versus excitation power with linear fitting. (**c**) SHG spectra of Cr_2_S_3_ nanosheets with different thicknesses under identical excitation conditions. Inset: extracted SHG peak intensity as a function of nanosheet thickness. (**d**) SHG intensity mapping of stacked Cr_2_S_3_ nanosheets. Inset: corresponding bright-field optical image. Scale bar: 20 μm.

We next measured the SHG signals from Cr_2_S_3_ nanosheets under various excitation powers. As shown in Figure 2a, prominent SHG signals are observed at 400 nm, with intensities monotonically increasing as the excitation power increases from 1.0 mW to 6.0 mW. The double-logarithmic plot of SHG intensity versus excitation power yields a slope of 1.8 ± 0.05 (Figure 2b), which is in good agreement with the theoretical quadratic dependence expected for a second-order nonlinear optical process [23], confirming that the observed signals originate from SHG. To identify the origin of SHG, we measured SHG spectra from rhombohedral Cr_2_S_3_ nanosheets of varying thicknesses. As shown in Figure 2c, the SHG intensity exhibits a monotonic increase with increasing sample thickness. All measured Cr_2_S_3_ thicknesses were determined by AFM (Supplementary Note S3). Furthermore, SHG mapping of stacked rhombohedral Cr_2_S_3_ flakes reveals a clear thickness-dependent enhancement of SHG signal intensity (Figure 2d). This thickness-dependent behavior stands in stark contrast to that of trigonal phase Cr_2_S_3_, where SHG intensity decreases with increasing thickness [21]. The physical origin lies in the intrinsically non-centrosymmetric bulk structure of rhombohedral Cr_2_S_3_, where strong covalent coupling between layers via face-sharing CrS_6_ octahedra ensures that the non-zero second-order nonlinear susceptibility *χ*^(2)^*_bulk_* permeates the entire sample thickness, rather than being confined to surface regions. In addition, the high crystalline quality and a favorable refractive index dispersion of rhombohedral Cr_2_S_3_ ensure a coherence length exceeding the measured thickness range, enabling SHG intensity with thickness.

**Figure 3:**
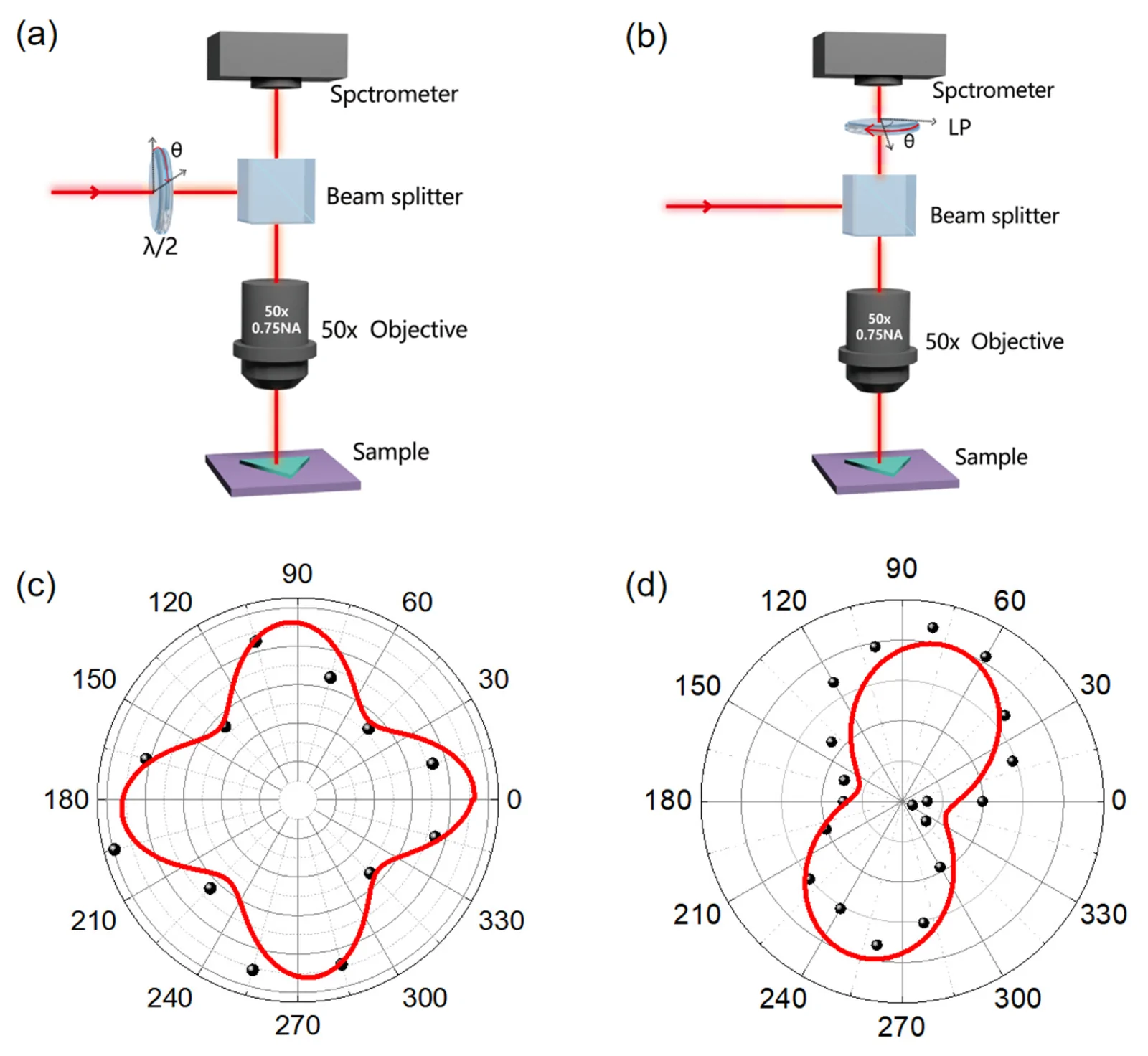
Polarization-resolved SHG measurement configuration and polarization-dependent SHG response of Cr_2_S_3_ nanosheets. (**a**,**b**) Experimental configurations for excitation and detection polarization-dependent SHG intensity measurements. (**c**,**d**) Polar plots of SHG intensity as a function of excitation and detection angles. The red curves follow the relation *I* ∝ *I*_0_ sin^2^(3*θ*), where *θ* is the angle between the laser polarization and the crystal mirror plane, and *I*_0_ is the maximum SHG intensity.

To elucidate the origin of the SHG signals, we performed polarization-dependent SHG measurements on rhombohedral Cr_2_S_3_ nanosheets by independently rotating the excitation and detection polarizations (Figure 3a,b). Figure 3c,d display the SHG intensity as a function of excitation angle (*θ_exc_*) and detection angle (*θ_det_*). When the detection polarization is fixed and the excitation half-wave plate is rotated to vary the incident polarization angle, the SHG intensity exhibits a distinct fourfold symmetry pattern with four intensity maxima near 0°, 90°, 180°, and 270°, and four minima near 45°, 135°, 225°, and 315°, with a maximum-to-minimum ratio of approximately 2:1. This fourfold pattern arises from the tensor coupling between the excitation and detection polarizations. When the excitation polarization is fixed and the detection polarizer is rotated, the SHG intensity exhibits highly linear polarization dependence, reaching a maximum when the detection polarization is parallel to the excitation polarization (XX configuration) and dropping to near-zero minimum when perpendicular (XY configuration), with a ratio exceeding 10:1. This highly linear polarization characteristic originates from the *χ*^(2)^ tensor structure dictated by the $R\bar{3}$ space group of rhombohedral Cr_2_S_3_. We also measured the SHG intensity as a function of sample rotation angle, where the polarization-dependent SHG exhibits six equal-intensity lobes (Supplementary Note S4), indicating threefold rotational symmetry, consistent with previous reports [9]. Moreover, because the rhombohedral phase possesses an intrinsically non-centrosymmetric bulk structure rather than relying solely on surface effects, the bulk nonlinear susceptibility tensor remains highly uniform across the entire sample thickness, avoiding polarization decoherence induced by surface/interface disorder and ensuring that the detection polarization-resolved SHG exhibits pronounced linear polarization selectivity.

**Figure 4:**
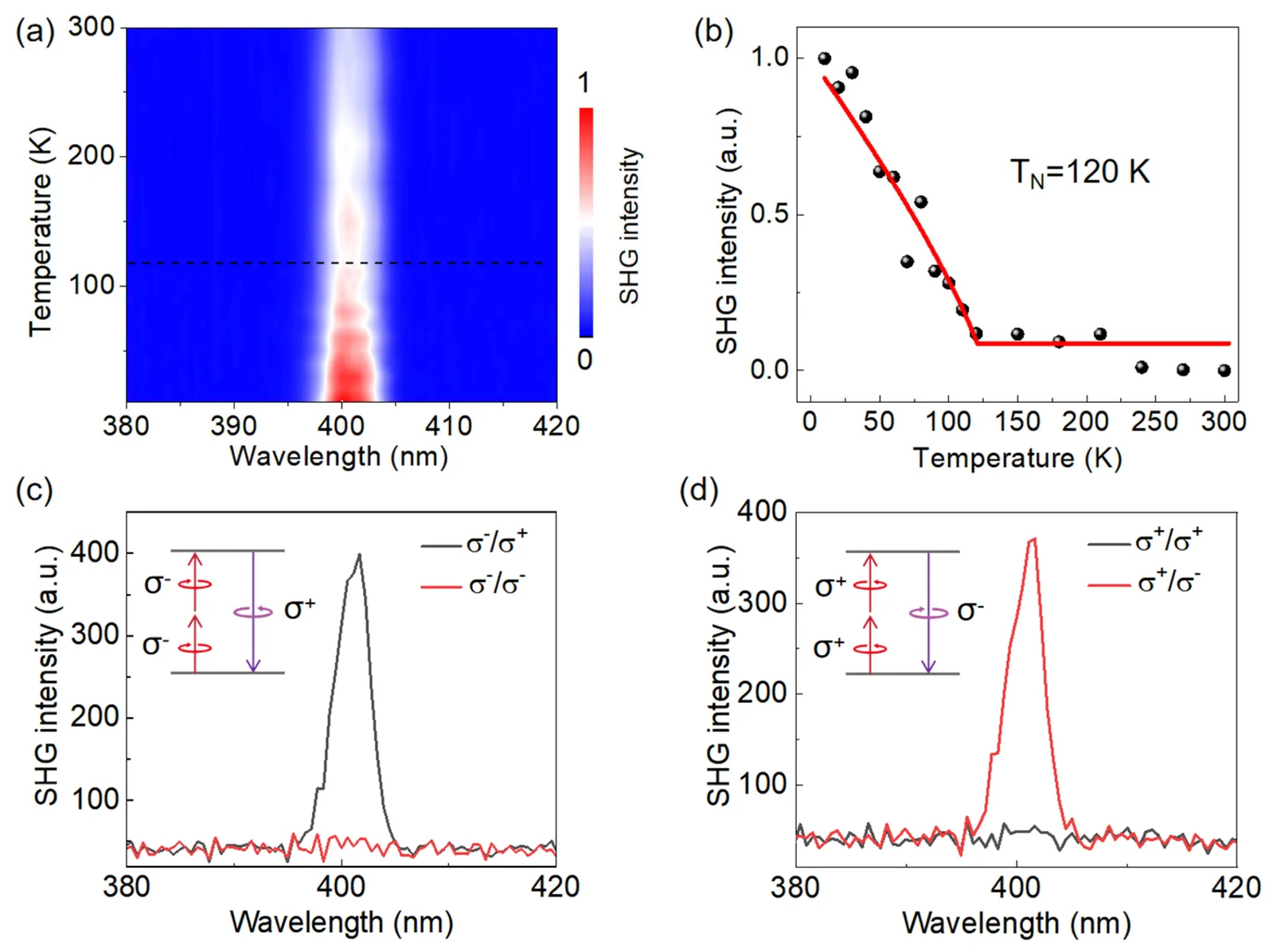
Temperature-dependent and circularly polarized SHG of Cr_2_S_3_ nanosheets. (**a**) Temperature-dependent SHG spectra of Cr_2_S_3_ nanosheets. (**b**) Normalized temperature-dependent SHG intensity extracted from (**a**). The red solid line is the fitting result following the power–law relation |1 − (*T*/*T_N_*) |^2*β*^ for T < T_N_. The fit results are *β* = 0.79 ± 0.15, with a confidence level of approximately 92%. (**c**,**d**) Circular polarization-resolved spectra excited with *σ*^−^ and *σ*^+^ at 10 K. Insets: optical selection rules for circularly polarized SHG. The upward and downward arrows denote the fundamental and SHG signals, respectively.

We further probed the magnetic ordering transition of Cr_2_S_3_ via temperature-dependent SHG measurements. As a non-van der Waals ferrimagnetic material, rhombohedral Cr_2_S_3_ exhibits a Néel temperature (T_N_) of approximately 120 K, below which a transition from paramagnetic to ferrimagnetic ordering occurs. Since SHG is highly sensitive to both spatial inversion and time-reversal symmetry, the emergence of magnetic ordering breaks time-reversal symmetry, leading to detectable changes in the SHG response [29,30]. In the temperature-dependent SHG experiments, as the temperature decreases from 300 K to 120 K (Figure 4a), the SHG intensity remains relatively stable, primarily originating from the temperature-independent lattice nonlinear response. When the temperature decreases below T_N_ ≈ 120 K, the SHG intensity exhibits a significant enhancement, which increases sharply upon further cooling to 10 K (Figure 4b). The evolution of the SHG intensity with temperature follows a power–law relationship |1 − (T/T_N_)|^2β^ (where T is temperature, T_N_ is the Néel temperature, and β is the critical exponent), which is characteristic of the evolution of magnetic ordering parameters. This enhancement is attributed to the establishment of magnetic order in Cr_2_S_3_ below the T_N_, which breaks time-reversal symmetry and activates the magnetic dipole SHG channels [29,30], whose intensity is proportional to the square of the magnetic order parameter and therefore increases sharply with cooling. Therefore, by monitoring the anomalous temperature-dependent variation in SHG intensity, the magnetic transition temperature of Cr_2_S_3_ can be precisely determined, providing access to critical magnetic structure information including magnetic ordering and spin reorientation.

We further investigated the nonlinear optical response under ferrimagnetic ordering via circularly polarized SHG measurements. As shown in Figure 4c, under *σ*^−^ laser excitation, the co-polarized (*σ*^−^ detection) and cross-polarized (*σ*^+^ detection) SHG signals exhibit a high degree of circular polarization. Correspondingly, under *σ*^+^ laser excitation, the SHG signals exhibit the opposite high degree of circular polarization (Figure 4d). The SHG circular polarization is defined as: *ρ* = (*I_σ_*^−^ − *I_σ_*^+^)/(*I_σ_*^−^ + *I_σ_*^+^), where *I_σ_*^−^ and *I_σ_*^+^ represent the detected SHG intensities under *σ*^−^ and *σ*^+^ excitation, respectively. This near-100% circular polarization demonstrates the nearly perfect circular polarization optical selectivity of the system, whose physical origin lies in the synergistic effect of reduced crystal symmetry and time-reversal symmetry breaking induced by magnetic ordering [29,30]. Under *σ*^−^ excitation, the SHG mapping of co-polarized (*σ*^−^ detection) and cross-polarized (*σ*^+^ detection) also exhibits significant intensity contrast (Supplementary Note S5), consistent with the circularly polarized SHG spectroscopy results. These results reveal the coupling mechanism between the magnetic ordered structure and the nonlinear optical process.

## 4. Conclusions

We have successfully achieved controllable synthesis of non-layered rhombohedral Cr_2_S_3_ nanosheets via a CVD strategy, with thicknesses tunable from approximately 3.5 nm to several tens of nanometers. Power-dependent SHG measurements confirm the intrinsic second-order nonlinear optical origin of rhombohedral Cr_2_S_3_, with intensity increasing with increasing thickness. Excitation polarization-resolved SHG exhibits a four-lobed pattern, while detection polarization-resolved SHG reveals highly linear polarization selectivity. Temperature-dependent SHG measurements reveal a distinct inflection point at T_N_ ≈ 120 K, establishing SHG as a sensitive all-optical probe for magnetic ordering. The discovery of near-perfect circular polarization selectivity (~100%) at low temperatures further highlights the strong coupling between magnetic order and nonlinear optical responses in this material. Compared to other Cr-based chalcogenides, such as the centrosymmetric Cr_2_Se_3_ or trigonal-phase Cr_2_S_3_ where SHG is predominantly surface-derived, rhombohedral Cr_2_S_3_ exhibits bulk-origin SHG with thickness-enhanced efficiency and near-perfect circular polarization selectivity. The strong and polarization-dependent SHG response of rhombohedral Cr_2_S_3_ facilitates promising applications in nonlinear frequency conversion, all-optical spintronics, and quantum photonics, while its compatibility with scalable CVD growth facilitates device integration. Future studies exploring heterostructures with other 2D materials or integrating Cr_2_S_3_ into photonic crystal cavities could further enhance the SHG efficiency and enable novel device functionalities. These findings establish rhombohedral Cr_2_S_3_ as a promising platform for nonlinear photonic applications and provide important guidance for the development of magneto-optic and quantum optical devices based on 2D magnetic materials.

## Data Availability

The original contributions presented in this study are included in the article/Supplementary Materials. Further inquiries can be directed to the corresponding authors.

## Supplementary Materials

The following supporting information can be downloaded at: https://www.mdpi.com/article/10.3390/nano16171088/s1, Note S1. Schematic of circularly polarized SHG measurement setup. Note S2. SEM Characterization of Cr_2_S_3_ nanosheets. Note S3. AFM characterization of Cr_2_S_3_ nanosheets. Note S4. Polarization-resolved SHG of rhombohedral Cr_2_S_3_ nanosheets. Note S5. Circularly polarized SHG mapping of Cr_2_S_3_ nanosheets.
