# Supplementary material for: Controllable Chemical Vapor Deposition Synthesis and Second-Harmonic Generation of Rhombohedral Cr2S3
Source: Nanomaterials (Basel). 2026 Aug 31;16(17):1088. doi: 10.3390/nano16171088 (PMC13567280; doi:10.3390/nano16171088)
Supplement: Supplementary file 1 [file nanomaterials-16-01088-s001.zip › nanomaterials-4511606-supplementary.pdf]

Supplementary Information

Controllable Chemical Vapor Deposition Synthesis and Second-Harmonic Generation of Rhombohedral Cr_2S_3

Danliang Zhang[1], Peiran Li[1], Sihan Liu[1], Qing Ye[2], Ying Chen[2]*

[1]Key Laboratory of Low-Dimensional Quantum Structures and Quantum Control of Ministry of Education, Hunan Research Center of the Basic Discipline for Quantum Effects and Quantum Technologies, School of Physics and Electronics, Hunan Normal University, Changsha 410081, China.

[2]State Key Laboratory of Pulsed Power Laser Technology, Key Laboratory of Electronic Restriction of Anhui Province, National University of Defense Technology, Advanced Laser Technology Laboratory of Anhui Province, Hefei 230037, P. R. China.

**Email: danliangzhang@hunnu.edu.cn*

Table of Contents:

Note 1. Schematic of circularly polarized SHG measurement setup

Note 2. SEM Characterization of Cr_2S_3 nanosheets

Note 3. AFM characterization of Cr_2S_3 nanosheets

Note 4. Polarization-resolved SHG of rhombohedral Cr_2S_3 nanosheets

Note 5. Circularly polarized SHG mapping of Cr_2S_3 nanosheets

Note S1. Schematic of circularly polarized SHG measurement setup

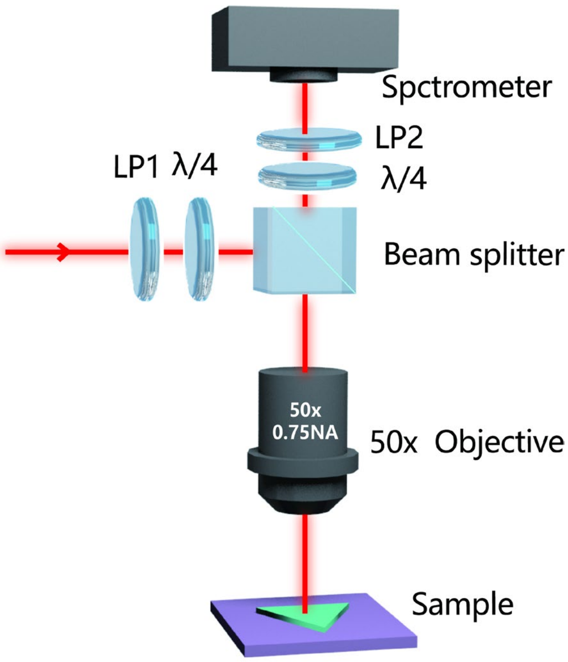

Figure S1. Optical schematic of the circularly polarized SHG measurement setup.

Note S2. SEM Characterization of Cr_2S_3 nanosheets

Scanning electron microscopy (SEM) combined with energy-dispersive X-ray spectroscopy (EDS) is a critical tool for characterizing the surface morphology and chemical composition of Cr_2S_3 nanosheets. We performed SEM-EDS characterization on CVD-grown Cr_2S_3 nanosheets to confirm their elemental composition and stoichiometric ratio. SEM image reveals flat, continuous, and uniformly covered Cr_2S_3 nanosheet surfaces without apparent cracks or voids (Figure S2a). The corresponding Cr and S element distribution mapping are shown in Figures S2b and 2c. EDS analysis (table inset) shows that only Cr and S elements are detected, confirming the high chemical purity of the product (Figure S2 d, e). Quantitative analysis indicates that the atomic percentages of Cr and S are 37.21% and 62.79%, respectively, with a ratio of Cr:S ≈ 1:1.69, which is in close agreement with the ideal stoichiometric ratio of Cr_2S_3 (2:3 = 1:1.5) [1]. The high-purity Cr_2S_3 nanosheets provide a reliable material foundation for subsequent nonlinear optical performance studies.

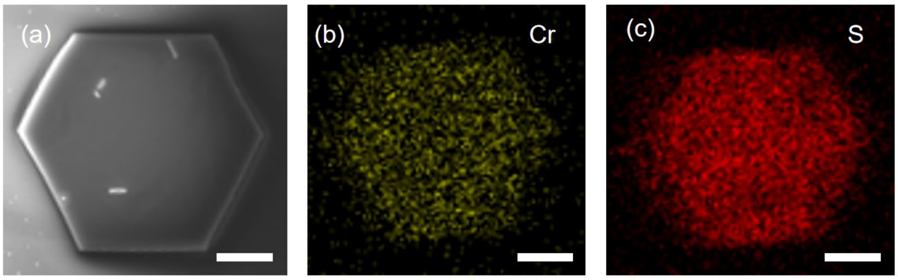

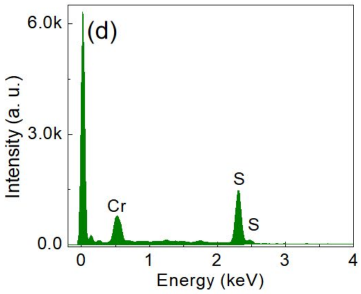

(e)

Element	Weight %	Atomic %
Cr	26.76	37.21
S	73.24	62.79
Totals	100.00	

Figure S2. SEM-EDS analysis of CVD-grown Cr_2S_3 nanosheets. (a) SEM image of Cr_2S_3 nanosheets and corresponding (b,c) Cr and S elemental mappings. Scale bar: 1 μm. (d) EDS spectrum of Cr_2S_3 showing only Cr and S characteristic peaks. (e) Weight and atomic percentages of Cr and S elements.

Note S3. AFM characterization of Cr_2S_3 nanosheets

To precisely determine the thickness of the CVD-grown few-layer Cr_2S_3 nanosheets, AFM measurements were performed on representative samples. As shown in Figure S3a–d, large-area AFM topography images reveal that rhombohedral Cr_2S_3 nanosheets of varying thicknesses exhibit flat surfaces with well-defined edges and uniform thickness across large areas. Quantitative analysis of the corresponding cross-sectional height profiles yields sample thicknesses of approximately 13 nm, 23 nm, 38 nm, and 60 nm, respectively (Figure S3 e–h).

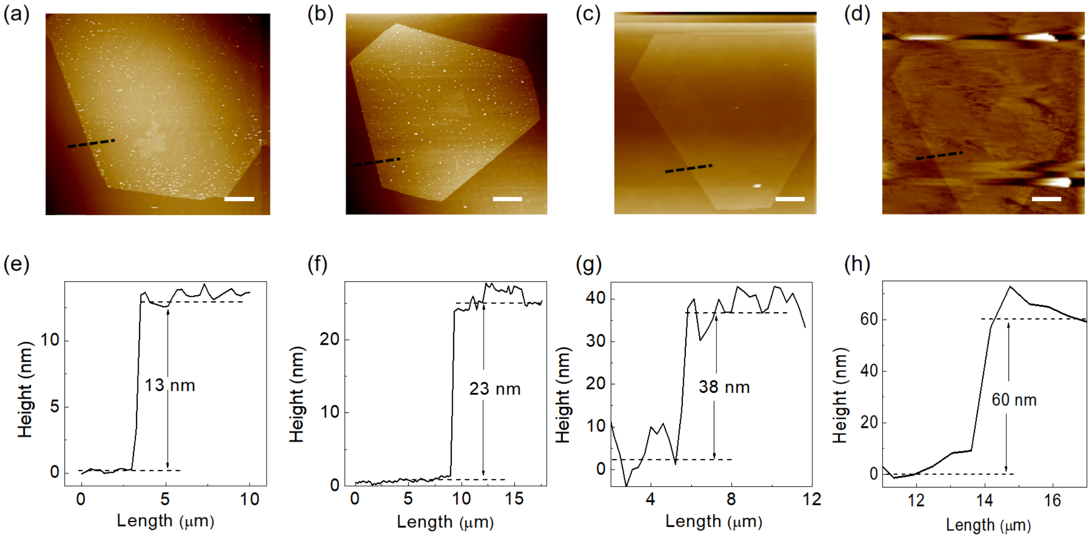

Figure S3. AFM characterization of representative Cr_2S_3 nanosheets. (a–d) AFM height topography images. Scale bar: 10 μm. (e–h) Corresponding height profiles along the black dashed lines.

Note S4. Polarization-resolved SHG of rhombohedral Cr_2S_3 nanosheets

To verify the symmetry of the CVD-grown Cr_2S_3 nanosheets, we measured polarization-resolved SHG signals. With the excitation and detection linear polarizations maintained in a perpendicular configuration, SHG signals were collected while rotating the rhombohedral Cr_2S_3 nanosheet in-plane. As shown in the figure S4, the SHG intensity as a function of the in-plane rotation angle (θ) exhibits a six-lobed polarization pattern with adjacent lobes separated by approximately 60°, indicating threefold rotational symmetry of the Cr_2S_3 lattice. Similar results have been reported for Cr_2S_3 nanosheets with $R\bar{3}$ space group [2].

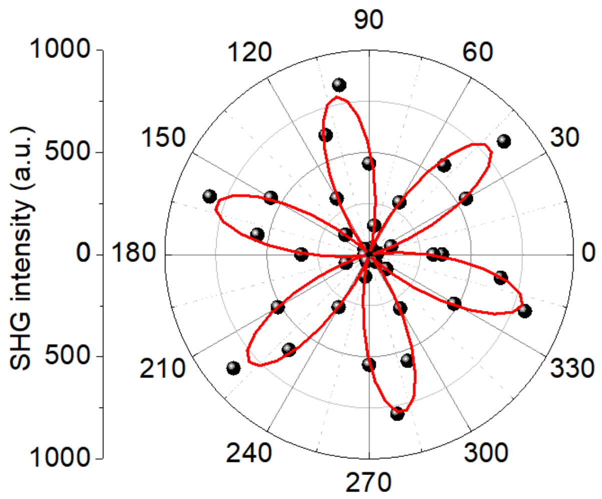

Figure S4. Polarization-resolved SHG polar plot of Cr_2S_3 nanosheets. The solid black dots represent the experimental data, while the solid curve is the fit following the relation $I = I_0 \cos^2(3\theta)$, where θ represents the azimuthal angle between the input laser polarization and the armchair direction of the crystal, and I_0 is the maximum SHG intensity in the perpendicular configuration.

Note S5. Circularly polarized SHG mapping of Cr_2S_3 nanosheets

We performed circularly polarized SHG spatial imaging measurements on CVD-grown rhombohedral Cr_2S_3 nanosheets to reveal the spatial distribution characteristics of magnetic ordering. Under left-handed circularly polarized (σ^-) excitation, the cross-polarized (σ^-/σ^+) and co-polarized (σ^-/σ^-) SHG mappings of rhombohedral Cr_2S_3 nanosheets exhibit near-100% intensity contrast (Figure S5 b,c), with the cross-polarized channel almost completely suppressed. This phenomenon originates from the chiral selectivity of the magneto-dipole process in the low-temperature magnetically ordered state.

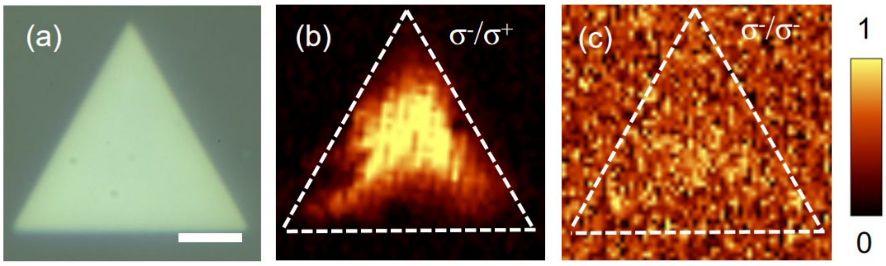

Figure S5. Circular polarization-resolved SHG spectra of Cr_2S_3 under the σ^- excitation. Optical image of (c) Cr_2S_3 and the corresponding normalized polarization-resolved SHG intensity mapping (b) σ^-/σ^+, (c) σ^-/σ^-. Scale bar: 10 μm.

References

1. Debnath, A.; Mondal, T.; Mahapatra, G.; Kapuria, A.; Saha, S. K., Observation of ferrimagnetic ordering in chemically synthesized 2D Cr_2S_3 nanosheets. *J. Appl. Phys.* **2023,** *133* (15).
2. Chu, J.; Zhang, Y.; Wen, Y.; Qiao, R.; Wu, C.; He, P.; Yin, L.; Cheng, R.; Wang, F.; Wang, Z.; Xiong, J.; Li, Y.; He, J., Sub-millimeter-Scale Growth of One-Unit-Cell-Thick Ferrimagnetic Cr_2S_3 Nanosheets. *Nano Lett.* **2019,** *19* (3), 2154-2161.
